# Defect Formation, T-Atom Substitution and Adsorption of Guest Molecules in MSE-Type Zeolite Framework—DFT Modeling

**DOI:** 10.3390/molecules26237296

**Published:** 2021-12-01

**Authors:** Petko St. Petkov, Kristina Simeonova, Iskra Z. Koleva, Hristiyan A. Aleksandrov, Yoshihiro Kubota, Satoshi Inagaki, Valentin Valtchev, Georgi N. Vayssilov

**Affiliations:** 1 Faculty of Chemistry and Pharmacy, University of Sofia, 1 James Bourchier Blvd., 1164 Sofia, Bulgaria; ohks@chem.uni-sofia.bg (K.S.); ohik@chem.uni-sofia.bg (I.Z.K.); haa@chem.uni-sofia.bg (H.A.A.); gnv@chem.uni-sofia.bg (G.N.V.); 2 Division of Materials Science and Chemical Engineering, Yokohama National University, 79-5 Tokiwadai, Hodogaya-ku, Yokohama 240-8501, Japan; kubota-yoshihiro-sr@ynu.ac.jp (Y.K.); inagaki-satoshi-zr@ynu.ac.jp (S.I.); 3 Laboratoire Catalyse et Spectrochimie, Normandie Université, ENSICAEN, CNRS, 6 Boulevard Maréchal Juin, 14050 Caen, France; valentin.valtchev@ensicaen.fr

**Keywords:** zeolite, silanol vacancy, Ti incorporation, MSE framework, DFT modeling

## Abstract

We used computational modeling, based on Density Functional Theory, to help understand the preference for the formation of silanol nests and the substitution of Si by Ti or Al in different crystallographic positions of the MSE-type framework. All these processes were found to be energetically favorable by more than 100 kJ/mol. We suggested an approach for experimental identification of the T atom position in Ti-MCM-68 zeolite via simulation of infrared spectra of pyridine and acetonitrile adsorption at Ti. The modeling of adsorption of hydrogen peroxide at Ti center in the framework has shown that the molecular adsorption was preferred over the dissociative adsorption by 20 to 40 kJ/mol in the presence or absence of neighboring T-atom vacancy, respectively.

## 1. Introduction

**Figure 1:**
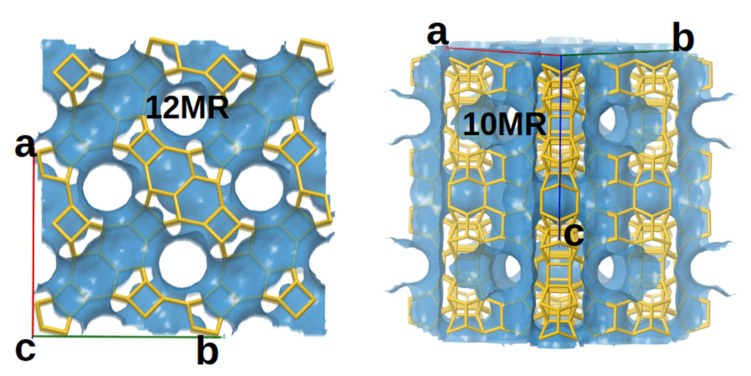
Schematic representation of the 12-membered and 10-membered channels of MSE-type zeolite. The figure is obtained frm the Animated Drawing of the MSE Framework, available on the IZA website (https://europe.iza-structure.org (accessed on 15 October 2021)).

The production of a variety of chemical compounds with industrial importance is possible with selective catalytic oxidation reactions. Hydrogen peroxide is one of the most successful oxidizing agents from the green chemistry point of view. A titanosilicate catalyst can activate H_2_O_2_ using selective oxidation to provide indispensable chemical resources [1]. Many studies demonstrated that titanosilicates with isolated Ti-centers in framework compounds show high efficiency toward the selective oxidation of organic molecules with hydrogen peroxide [2,3,4,5,6]. The first titanosilicate catalyst, which is industrially used for phenol oxidation, is Titanium silicate -1 (TS-1) [2,7,8]. TS-1 has an MFI-type framework with a system of crossing straight and sinusoidal 10-Membered Ring (10-MR) channels, which are able to accommodate the molecules with the size of the phenol. Still, the molecule diffusion, as well as the diffusion of the oxidation products, is limited. Recently, a new zeolite material Ti-MCM-68 [9] with an MSE-type framework was proposed for phenol oxidation catalysts with high activity and selectivity toward hydroquinone formation. The MSE framework offers a 12 × 10 × 10-ring (12 × 10 × 10-MR) pore system (shown on Figure 1). In this material, a straight 12-MR channel intersects two independent tortuous 10-MR channels. This structure also contains an 18 × 12-MR supercharge accessible only through the 10-MR channels present [10]. The Ti-MCM-68 material with MSE-type framework exhibits a large number of T-atom vacancies, in which the position of the missing Si Tetrahedral atom (T-atom) is occupied by a silanol nest. Besides the high catalytic activity and selectivity of Ti-MCM-68 zeolite, the stability of different Ti positions combined with the presence of defects (silanol nests) in the framework is still not well studied.

To contribute to the understanding of the properties of Ti-MCM-68 material and its comprehensive characterization, here we present an estimate for the relative stability of the silanol nest and substitution of Si by Ti or Al in different crystallographic positions of the framework using Density functional theory (DFT). We also estimated the effect of the presence of silanol nest in neighboring T-atom positions next to Ti center, which explains the important role of the silanol nest for the stabilization of the MSE-framework upon isomorphous substitution of Si by Ti. Furthermore, to check if the infrared spectra of adsorbed probe molecules may be used for the determination of Ti position in Ti-MCM-68 material, we modeled adsorption complexes of pyridine and acetonitrile to the Ti center incorporated at all accessible crystallographic positions in the MSE framework. Finally, adsorption of hydrogen peroxide at Ti center in the framework was modeled both in the absence and in the presence of neighboring T-atom vacancy.

## 2. Results and Discussion

### 2.1. Formation of Si Defects in MSE-Type Framework

**Figure 2:**
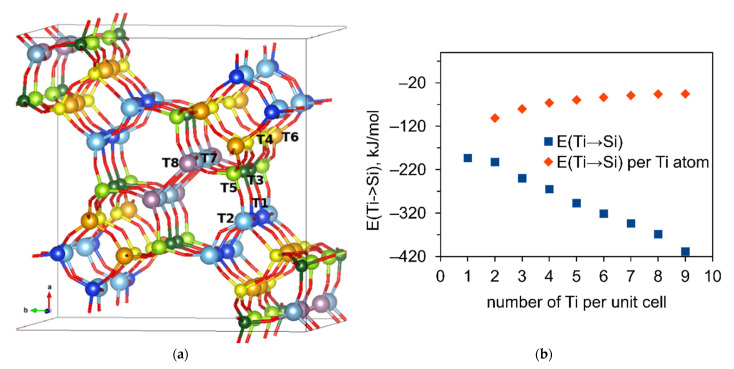
(**a**) Colored representation of the different T-atom sites in the MSE-framework. (**b**) Substitution of Si by Ti, rhombus show the substitution energy calculated by the following equation Zeo[(SiO_2_)_112_] + nTi(OH)_4_ → Zeo[(SiO_2_)_112-n_(TiO_2_)n] + nSi(OH)_4_, the squares show the same energy but divided by the number of the Ti in the unit cell.

Incorporation of Ti in tetrahedral position in the zeolite framework is possible both during the zeolite synthesis and via post-synthetic isomorphic substitution of Si by Ti. The second approach involves the formation of a framework vacancy prior to the incorporation of a substituting atom. In order to check the preference in the substitution of Si in the MSE-type structure, we determined the relative stability of T-atom vacancies formed by the removal of the selected T-atom, resulting in the formation of a silanol nest on its position. We considered the removal of Si from all 8 different crystallographic T-atom positions in the periodic structures with composition Si_112_O_224_. Different T-atom cites in MSE-type framework are noted in Figure 2.

To estimate the energy for Si vacancy formation, we considered formally the hydrolysis of four Si-O bonds in the framework and, thus, the formation of silanol nest according to the following formal reaction:

Zeo[(SiO_2_)_112_] + 4nH_2_O → Zeo[Si_112-n_O_224_H_4n_] + nSi(OH)_4_, (1)

where n is the number of the silanol nests.

The energies for the formation of single silanol nests in T1-T8 positions in the unit cell presented in Table 1 suggest that MSE-type framework shows high potential for Si-vacancy formation, as the formal process is exothermic. The calculated energies for Si-vacancy formation are in the range of −154 to −196 kJ/mol as the most favorable positions for silanol nests formation are positions T1, T4, T6, and T8 with essentially the same stability, followed by T2. The least favorable position of the vacancy is calculated to be T7. If we consider two silanol nest formations at the same time in T7 and T8 sites, as reported in an experimental work [11], the process is also energetically favorable. The energy for T7T8 silanol nest formation is −211 kJ/mol for neighboring defects, and −189 kJ/mol for distant silanol nests, i.e., the neighboring silanol nests formation is preferable. The same trend was observed for silanol nests formation at T3T5 positions, adjacent to T7T8, −257 kJ/mol, and −215 kJ/mol for neighboring and distant silanol nests, respectively. The calculated energy for double silanol nests formation at T7T8 and T3T5 suggests that T3T5 double silanol nest formation is more stable with respect to T7T8 by 46 kJ/mol. All these results are consistent with the hypothesis in our previous catalytic work [12]. Note that we are using T-site numbers according to IZA, which are different from those in Ref. [11].

### 2.2. Substitution of Si by Ti or Al

We also considered the substitution of Si by Ti or Al in all different 8 T-positions. The experimental Si/Ti ratio is in the range of 90 [9]. The unit cell of the MSE-zeolite framework contains 112 Si; thus, we started with a single Ti→Si substitution according to the following formal reaction:

Zeo[(SiO_2_)_112_] + Ti(OH)_4_ → Zeo[(SiO_2_)_111_(TiO_2_)] + Si(OH)_4_. (2)

For the substitution of Si for Al, the following formal reaction was considered:

Zeo[(SiO_2_)_112_] + Al(H_2_O)(OH)_3_ → Zeo[(SiO_2_)_111_(AlO_2_H)] + Si(OH)_4_ (3)

Since there are four different positions of the proton in the Al-O(H)-Si moiety, the relative stability of all four bridging OH groups for Al in each T site were considered, and for each T-site the one with the lowest energy was taken as a reference aluminosilicate structure.

In accordance with the favorable formation of silanol nest in all T-atom positions, the substitution of Si by Ti is also an energetically favorable process. The energy gain upon isomorphic substitution of Si by Ti varies between −160 and −193 kJ/mol, in the same energy range as the energy for silanol nest formation. The most stable structure is with Ti in T1 position, followed by T6, T2 and T4, which are by 14–19 kJ/mol less stable, and T8, T7, T5 and T3, 25–33 kJ/mol less stable. In Table 1, we also provide the calculated energy values for the incorporation of Ti in the position of silanol nest and restoring the zeolite framework. For most T sites the process is slightly endothermic with the highest value for T8 site, 20 kJ/mol. For T1 site, it is essentially energy neutral, −1 kJ/mol, and for T7 the process is slightly exothermic by −11 kJ/mol. The small absolute values of those energies suggest that in the real material, one may have structures with T-atom vacancy or with Ti with similar stability.

Since the substitution of Si by Ti is an energetically favorable process, we calculated a step-wise substitution of up to 9 Si by Ti only in the most stable T1 sites in the unit cell (up to Si/Ti ratio 11). The results are shown in Figure 2b. The substitution of Si by Ti leads to continuous stabilization of the zeolite structure with the increasing amount of Ti included in the framework. The energy for substitution of Si by Ti normalized to the number of Ti atoms included in the unit cell converges to −40 kJ/mol per Ti after 7 Ti atoms per unit cell. These results show the MSE framework’s potential to accommodate a higher amount of Ti.

Although for TS-1 zeolite, all results based on energetic stability of the framework due to the isomorphic substitution of Si by Ti, show no preferable position of Ti, an experimental study suggested that some T-sites are more populated by Ti over others [14]. A possible reason for such discrepancies in the theory and experiment may be the formation of T-atom vacancy/silanol nest in the vicinity of the Ti, which may lead to stabilization of some of the Ti-sites. In order to check the preference of the Ti-MSE framework to form additional silanol nests in the vicinity of the Ti sites, the energy for silanol nest formation was calculated in Ti-MSE framework with one Ti in the framework for all T sites exposed to the channels of the MSE structure, T1 to T6 (Table 2). For each structure, a silanol nest was created in each of the neighboring T-atom positions.

**Figure 3:**
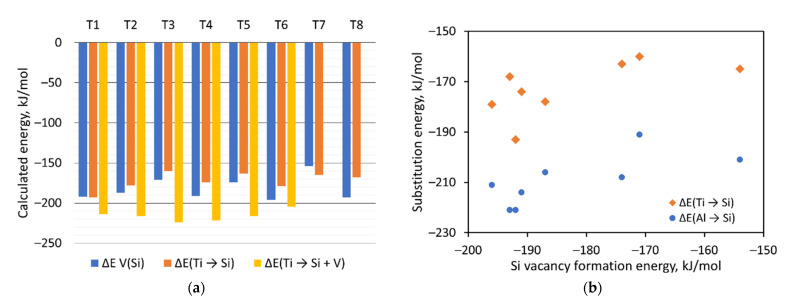
(**a**) Calculated energies for Si vacancy formation, the substitution of Si by Ti, and substitution of Si by Ti with simultaneous formation of neighboring Si vacancy (the most stable location of the vacancy was used). (**b**) Substitution energies for Si by Al or Si by Ti versus Si vacancy formation energy at the corresponding T-atom position.

According to the results listed in Table 2, the process of silanol nest formation is energetically favorable around all Ti, with some exceptions around Ti in T1 position. The energy for silanol nest formation varies from +1 kJ/mol (for Ti in T1 and silanol nest in T2) to −63 kJ/mol (for Ti in T3 and silanol nest in T4 position). For comparison, the same process was calculated for the MFI-framework, on which is based TS-1 catalyst. In the MFI framework, with one Ti in T7 position, the silanol nest formation in the adjacent position of Ti is also an energetically favorable process in three T-atom positions, and energetically unfavorable in one T-atom position. It seems that silanol nest formation in the vicinity of the Ti leads to relaxation of the Ti-O bonds to the optimal value due to the reduced local framework strain. For instance, the Ti-MSE, with Ti in T3 position without defects in the vicinity of the Ti, show four Ti-O distances of 1.81, 1.80, 1.81, and 1.79 Å. Upon the formation of silanol nest in the T4 position, supported with an energy gain of 63 kJ/mol, the corresponding Ti-O distances relax to 1.82, 1.84, 1.82, and 1.80 Å, closer to the optimal value of 1.83 Å, which is calculated for isolated cluster model Si(OH)_3_-O-Ti(OH)_3_ using the same computational protocol. Thus, the ability of the framework to form silanol nests in the vicinity of the Ti is important for stabilizing the zeolite structure (Figure 3a).

We also modeled substitution of Si by Al with simultaneous formation of a bridging hydroxyl group. The substitution energy varies between −191 and −221 kJ/mol, i.e., by about 30 kJ/mol more exothermic than the substitution of Si by Ti. The highest energy preference for Si substitution by Al is found for T1 and T8 positions, while at T3 position, the substitution is the least exothermic. No clear correlation was found between Si vacancy formation energy and energy for Si substitution by Al or by Ti (Figure 3b).

### 2.3. Adsorption Complexes of Pyridine and Acetonitrile on Ti Centers

**Figure 4:**
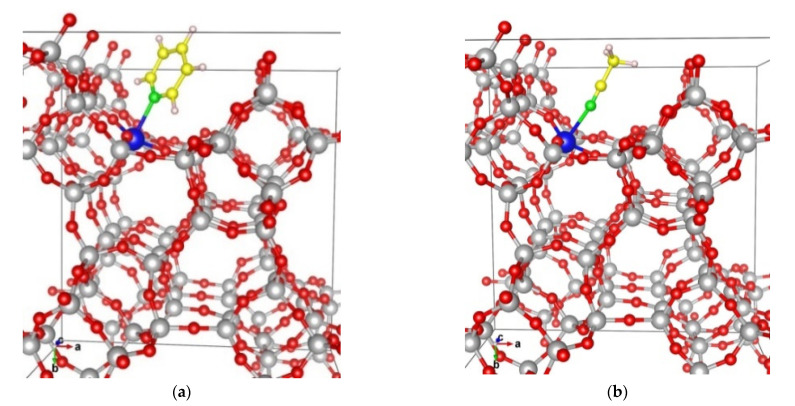
Adsorption complexes of pyridine (**a**) and acetonitrile (**b**) on Ti centers incorporated in position T1 of MSE type zeolite. Color coding: Si—gray, Ti—blue, O—red, N—green, C—yellow, H—white.

We modeled adsorption complexes of pyridine and acetonitrile at Ti^4+^ cations incorporated in T1, T2, T3, T4, T5, and T6 positions of MSE-type zeolite, which are exposed to the channels of the zeolite (Table 3, Figure 4). The complexes with the former ligand are more stable than the latter one, as the corresponding binding energies are −124 ÷ −95 kJ/mol and −72 ÷ −49 kJ/mol, respectively. We also calculated the Gibbs free binding energies at 298 K (ΔG) which are by 29 ÷ 40 kJ/mol and 25 ÷ 34 kJ/mol less exothermic than the corresponding BE values for pyridine and acetonitrile adsorption, respectively. As a trend, the BE values correlate with the Ti-N distances as the most stable complexes were formed with Ti^4+^ cation incorporated in position T6 for both pyridine and acetonitrile ligands where the shortest Ti-N distances were also found: 231 and 232 pm, respectively. On the other hand, the least stable complexes were calculated with Ti centers located in the T3 position in the case of pyridine and T4 and T2 positions for acetonitrile. In these cases, the longest Ti-N contacts were found, 239, 241, and 245 pm, respectively.

The IR vibrational frequencies of the ligands in both types of complexes were also calculated. The C-N vibrational frequency of acetonitrile is shifted by 28–44 cm^−1^ to higher frequencies with respect to the values in the gas phase molecule, 2297 cm^−1^. The highest and lowest shifts were found for the most stable and one of the least stable complexes, where Ti is located in positions T6 and T2, respectively. The IR frequencies for the gas phase pyridine molecule in the range 1400–1600 cm^−1^ were calculated at 1567, 1565, 1454, and 1420 cm^−1^. When the adsorption complexes were formed, the corresponding frequencies were shifted by 22 ÷ 44, −4 ÷ 9, 12 ÷ 18, and 4 ÷ 13 cm^−1^, respectively. Similar shifts (32, 0, 10, and 17 cm^−1^, respectively) were found previously for the adsorption of pyridine at [W^VI^O] species incorporated in MFI type zeolite [15], though the binding energy in this case, −57 kJ/mol, is more than twice lower with respect to the most stable complexes in the present study.

We also modeled the adsorption of both organic molecules on Ti center in T6 position located close to a silanol nest, T6_pyr_vac, and T6_CH_3_CN_vac structures. The calculated IR frequencies are very similar for the structures with and without silanol nest in the vicinity of the Ti center (T6_pyr_vac vs. T6_pyr and T6_CH_3_CN_vac vs. T6_CH_3_CN). Hence, the presence of silanol nest does not influence notably the IR features of the adsorbates in the investigated Ti-containing zeolite system.

### 2.4. Adsorption of Hydrogen Peroxide at Ti in Ti-MSE in Presence or Absence of Si Vacancy

Hydrogen peroxide is an environmentally friendly oxidant because it can oxidize organic compounds with the generation of water only as a coproduct of the reaction and is also less expensive than organic peroxides. Many Ti-substituted molecular sieves with isolated Ti sites were studied for selective oxidation reaction with hydrogen peroxide [3,4,6,7,16]. We have focused our study only on Ti, which is rather incorporated in the framework than grafted on the surface of the zeolite channels. This assumption was provoked by the fact that Ti grafting inside the micropores prevents mass-transfer in the micropores and increase the hydrophilicity. Thus, we expect that grafted Ti ions will reduce the catalytic activity of the Ti-MSE material towards oxidation of bulky molecules with hydrohobic motifs as phenol in a contradiction to the results from Ref. [12].

**Figure 5:**
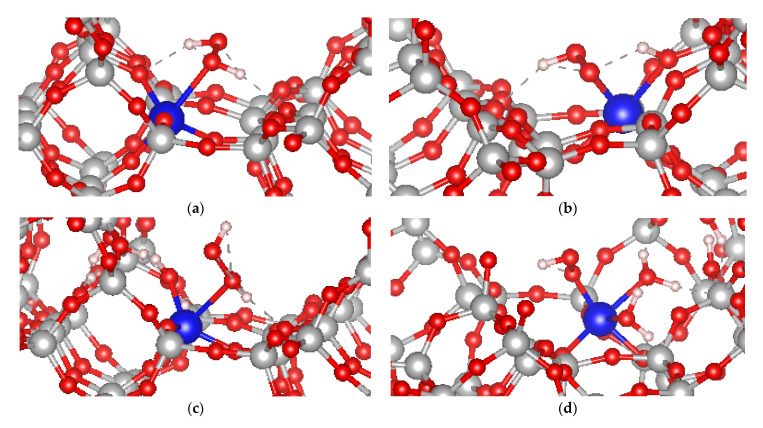
Non-dissociative (**a**,**c**) and dissociative (**b**,**d**) adsorption complexes of H_2_O_2_ at Ti centers in T1 position in MSE framework without (**a**,**b**) and with (**c**,**d**) T-atom vacancy in the vicinity of the Ti. The black arrows indicate the position of the proton from the H_2_O_2_ after dissociation.

To gain an insight into the interaction of hydrogen peroxide with the active sites of Ti-MSE zeolite, which is a highly selective catalyst for phenol oxidation by H_2_O_2_, we modeled four absorption complexes of H_2_O_2_ at Ti in T1 position in MSE-framework (Figure 5). We have studied dissociated and non-dissociated adsorption complexes of H_2_O_2_. The non-dissociative adsorption of H_2_O_2_ at a regular four-coordinated Ti site leads to the binding energy of −68 kJ/mol, the H_2_O_2_ molecule is bound via one of its O atoms to the Ti at a distance of 2.40 Å, while the two protons participate in H-bonds with O atoms from the zeolite framework at distances of 1.90 and 1.96 Å. Upon hydrogen peroxide dissociation, one of the protons of H_2_O_2_ is transferred to a framework O atom forming Ti-O(H)-Si moiety. This process is less energetically favorable as the binding energy for such dissociative adsorption of H_2_O_2_ decreases (in absolute value) to −23 kJ/mol, i.e., three times lower than the non-dissociative adsorption of H_2_O_2_. The higher stability of the non-dissociative adsorption of hydrogen peroxide confirms the results for an earlier computational study with isolated models, where the dissociative adsorption was found to be by about 30 kJ/mol disfavored [17]. The catalytic activity of the non-dissociatively adsorbed hydrogen peroxide as an oxidant was confirmed by Katz et al. [18] using model catalytic systems.

The energy of this process changes when in the vicinity of the Ti-site and a silanol nest is formed. In this case, the Ti may coordinate one H_2_O water together with the H_2_O_2_ and thus form a hexa-coordinated complex (Figure 5c,d). The binding energy for non-dissociative adsorption remains essentially the same as in the previous model without neighboring silanol nest, −66 kJ/mol. On the other hand, the binding energy for dissociative adsorption, −40 kJ/mol, suggests that the dissociation becomes more exothermic in comparison to the Ti without silanol nest in the vicinity. Thus, the silanol nest in the vicinity of Ti in MSE-framework stabilizes the Ti by reducing the local strain and facilitates the dissociation of hydrogen peroxide to form hydroperoxo species on Ti, similarly to earlier reports for TS-1 zeolite [19,20].

**Figure 6:**
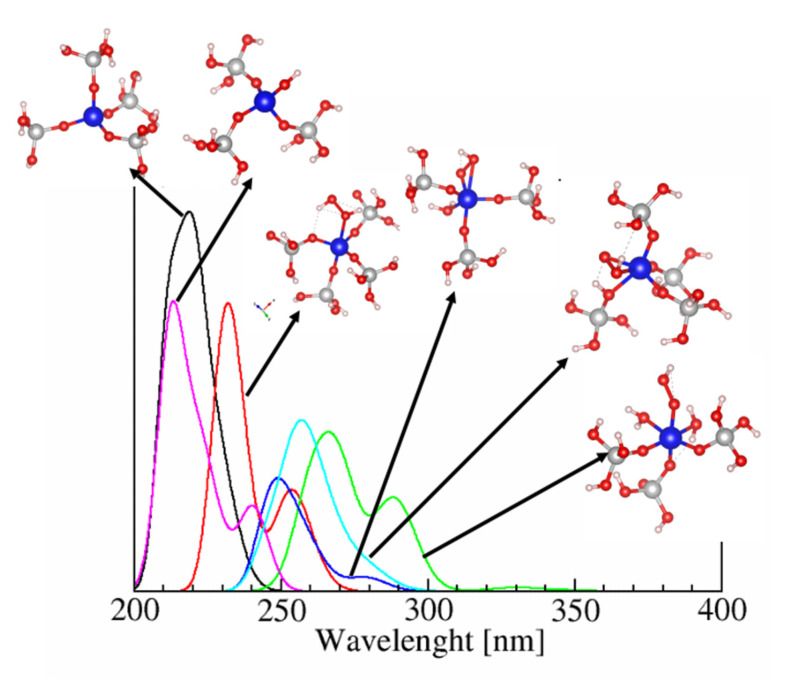
Calculated UV-vis spectra for isolated cluster model cut from the periodic model of Ti-MSE zeolite.

**Figure 7:**
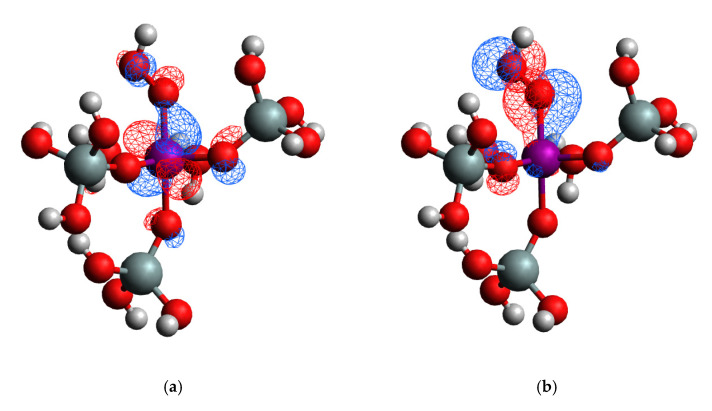
(**a**) LUMO and (**b**) HOMO for the complex of six coordinated Ti with dissociated H_2_O_2_ cut from the respective optimized periodic model.

The calculated UV-vis spectra for different cluster models representing 4, 5, and 6-coordinated Ti, with and without H_2_O_2_ adsorbed on it (Figure 6), in accordance with the literature data, [12] show that in the spectra of 5- and 6-coordinated Ti appears a second group of peaks in the 250–300 nm region. These peaks are associated with the formation of Ti-OOH hydroperoxo species. The analysis of the molecular orbitals shows that HOMO is localized mainly at the peroxo-part, while the LUMO is more localized over the Ti. That means the lowest energy transition (HOMO-LUMO transition) involves the peroxo part of the complex (Figure 7). This is the reason why the H_2_O_2_ adsorption leads to such a bathochromic shift in the UV-vis spectra of 5- and 6-coordinated Ti in comparison to the 4-coordinated Ti from the framework.

## 3. Discussion

In the experimental reports by the group of Kubota [11,21], the precursor YNU-2P, which is used for the synthesis of the MSE-zeolite framework, has significant site defects initially at the T7 and T8 sites. They hypothesize that further rearrangement of the silanol nests is possible by the steaming process. Our DFT data for silanol nests formation confirms that T8 is among the most favorable site for silanol nest formation. T8 position does not face the 12R channel, but it might migrate to its adjacent T5 position on the surface of the 12R channel, which is only by 13 kJ/mol less stable, thus contributing to the formation of available catalytic sites on the surface of the 12R channel. Our results suggest that the formation of double vacancies in neighboring T7 and T8 positions is also favorable. Moreover, it is more stable than those vacancies located distantly. The experimental evidence [9,11,21] that the silanol nests exist in T7 and T8 in the parent material and upon steaming migrate to their adjacent T3 and T5 position at the surface of the 12R channel is supported by our DFT results. We predict that T3T5 silanol nests are energetically more stable than T7T8 by 45 kJ/mol. Due to the lack of detailed atomistic simulations in the literature explaining such silanol nest migrations, our attention in future studies will be focused on the kinetics of the process. We are aiming to study the free-energy surface of silanol nest migration with non-equilibrium molecular dynamic simulations.

In accordance with the favorable formation of silanol nest in all T-atom positions, the substitution of Si by Ti is also an energetically favorable process. Earlier quantum chemical calculations for TS-1 zeolite [13,22,23] suggest that substituting Si by Ti resulted in very similar stability of structures with Ti in the different T-sites as the differences range up to 10–16 kJ/mol. The energy gain for substitution of Si by Ti in MSE framework, however, is calculated notably higher (in absolute values) in comparison to the Ti incorporation in the Si form of MOR and ITQ-44 frameworks, −14 and −59 kJ/mol [24], respectively, and also to TS-1, about −90 kJ/mol [13].

Such a high, in absolute value, energy for substitution of Si by Ti shows the potential of the MSE-framework as high Ti-containing material. Our further investigation found a possible synergism between the ability of the framework for silanol nests formation and Ti content in the framework. We have considered the formation of silanol nests in the vicinity of Ti in T1-T6 positions, because they are facing the surface of the 12R channel of the framework and thus are accessible for the adsorbates. Silanol nest formation in the vicinity of the Ti ions was found to be an energetically favorable process. Such defects actually reduce the local strain in the Ti ion structure, allowing the Ti-O bonds to their equilibrium state. Such kind of Ti site located near a silicon vacancy, terminated with hydrogen atoms forming a silanol nest, has been suggested for TS-1 by DFT modeling of isolated cluster models [19]. Here, on the basis of fully periodic models, we observe the same trend for Ti-MSE framework. Note, however, that the presence of Ti reduces the exothermicity of the silanol nest formation strongly in comparison with the pure Si structure, for which the energy for the formation of a vacancy was in the range −153 to −193 kJ/mol. To extend our study in the hypothesis that the vacancy formation near Ti ions may be responsible for the stability of the Ti centers and their catalytic performance in oxidation reaction with H_2_O_2_, we calculated the UV-vis spectra for various cluster models representing 4, 5, and 6-coordinted Ti, with and without H_2_O_2_ adsorbed on it (Figure 6). Our calculations confirm the literature data [12] that in the spectra of 5- and 6-coordinated Ti appears a second group of peaks in the 250–300 nm region, and the molecular orbital analysis (Figure 7) show that these peaks are associated with the formation of Ti-OOH hydroperoxo species, which are assumed to be catalytically active. In addition to the UV-vis spectroscopy, we tried to use the IR fingerprint of probe molecules as pyridine and acetonitrile to identify the position of the Ti in the framework or formation of defect near the Ti ion. Such methodology was used previously for adsorption of pyridine at [W^VI^O] species incorporated in MFI type zeolite [15]. The calculated vibrational frequency shifts of the characteristic bands of both molecules may be used to identify the position of Ti in MSE framework, at which the probe molecule is adsorbed only if the population of some of the T-sites is large enough to avoid an averaged convolution of the bands from all different T-sites. However, these probe molecules are not sensitive to the presence of silanol groups located in the vicinity of the Ti center.

## 4. Computational Details

We performed periodic DFT calculations using the PBE exchange-correlation functional with dispersion correction (PBE-D2) [25,26]. Vienna ab initio simulation package (VASP) [27,28], was employed for these calculations. The large size of the unit cell (see below) allowed us to sample the Brillouin zone using the Γ point only [29]. A plane-wave basis was used with a cut-off energy of 750 eV for the ion positions, cell shape, and size optimization, while for the adsorption energies of the probe molecules (cell volume and shape are fixed) the cut-off was reduced to 450 eV. The unit cell of the MSE framework consists of 112 T atoms and 224 oxygen atoms. It was optimized for the pure silicate structure with dimensions: a = 18.082 Å, b = 18.227 Å, c = 20.371 Å; α = β = γ = 90°. All atoms were allowed to relax until the force on each atom was less than 5 × 10^−2^ eV/Å during the geometry optimization. In all cases of vacancy formation or T-atom substitution, the cell shape and size were optimized together with the ionic positions, while for the adsorption calculations, only the ion positions were optimized. Vibrational frequencies of the probe molecules were calculated numerically. All calculations of the molecular species were performed in a gas phase and single molecule absorption was considered.

The reported energy values correspond to electronic energy differences as negative value corresponds to a more stable structure.

The UV-vis spectra were calculated with Tamm-Dankoff approximation [30] of the time-dependent density functional theory using hybrid exchange-correlation functional B3LYP [31,32,33,34] and triplet-zeta basis DEF2-TZVP [35]. The SCF convergence was achieved when the energy change was smaller than 1.0 × 10^−7^ a.u. The isolated cluster models were cut from the periodic models, and dangling bonds were saturated with hydrogen atoms. In the resulting clusters, only the position of the capping hydrogen atoms were optimized. The UV-vis calculations were performed with ORCA program [36].

The enthalpy and the entropy contributions to the Gibbs free energies were calculated at T = 298 K and partial pressure of 1 atm (101325 Pa). The enthalpy (H) of gas-phase pyridine and acetonitrile molecules is defined as the sum of total electronic energy (E_el_), internal vibrational energy (E_v_), zero-point vibrational energy (ZPVE), as well as the rotational and translational contributions to the internal energy: H = E_el_ + E_v_ + ZPVE + E_R_ + E_t_. The corresponding calculated entropies (S) includes translational, (S_t_) vibrational, (S_v_) and rotational (S_r_) degrees of freedom: S = S_t_ + S_v_ + S_r_. All the considered systems are in singlet state, thus the electronic contribution to the entropy is zero. Since both adsorbed molecules are constrained in the zeolite cavity their entropy contributions in the adsorption complexes were corrected as follows: the translational and rotational degrees of freedom were reduced by 1 and 1.28 [37]. The Gibbs free energies of the zeolite support consist of only vibrational and electronic contributions. The expressions of all enthalpy and entropy contributions can be found in Ref. [38].

## 5. Conclusions

By means of DFT calculations using a periodic model of MSE framework, we show that the formation of silanol nest is exothermic by more than 150 kJ/mol per silicon vacancy in different T-atom positions of the structure. It has also been observed that double silanol nests formation is an energetically favorable process for T3T5 and T7T8 sites. The easy Si vacancy formation facilitates the post-synthetic substitution of Si by Ti, which is also an energetically favorable process by 160 to 190 kJ/mol. In addition, the easy silanol nest formation in the vicinity of the Ti leads to stabilization of the Ti due to relaxation of the Ti-O bonds, which are by ~0.2 Å longer than Si-O bonds.

The adsorption of basic probe molecules, pyridine, and acetonitrile, at Ti sites of Ti-MSE zeolite suggests that it is strongest at T6, followed by T5 position of Ti. The calculated vibrational frequency shifts of the characteristic bands of both molecules may be used for the identification of the position of Ti at which the probe molecule is adsorbed. However, these probe molecules are not sensitive to the presence of silanol groups located in the vicinity of the Ti center.

The adsorption of hydrogen peroxide at Ti in Ti-MSE was found to be non-dissociative due to the higher exothermicity of the process by 45 kJ/mol compared to dissociative adsorption. Furthermore, the presence of silanol nest in the vicinity of Ti reduces the energy difference between the two types of complexes to 25 kJ/mol, thus facilitating the formation of hydroperoxo moieties.

## Figures and Tables

**Table 1:** Calculated relative energies (in kJ/mol) for silanol nest formation (ΔE V(Si)), Substitution (ΔE) of the Si atom for Al or Ti in all crystallographic T-atom positions in the Si form of MSE- framework.

T-Sites	∆E V(Si)	∆E(Ti → Si)	∆E(Ti → Si vac)	∆E(Al → Si)
T1	−192	−193	−1	−221
T2	−187	−178	9	−206
T3	−171	−160	11	−191
T4	−191	−174	17	−214
T5	−174	−163	11	−208
T6	−196	−179	17	−211
T7	−154	−165	−11	−201
T8	−193	−168	20	−221
T7T8 next	−211			
T7T8 far	−189			
T3T5 next	−257			
T3T5 far	−215			
MOR		−14		
ITQ−44		−59		
MFI [13]		~−90		

**Table 2:** Calculated relative energies for silanol nest formation (ΔE in kJ/mol) in the vicinity of Ti atom in Ti-MSE framework for the T-position on the surface of the 12-MR channel. The Si-vacancy formation energy was calculated according to the following formal process.

Zeo[(SiO_2_)_111_(TiO_2_)] + 4H_2_O → Zeo[(SiO_2_)_110_(TiO_2_)H_4_] + Si(OH)_4_								
Silanol Nest Position, ∆E [kJ/mol]								
Ti Position	T1	T2	T3	T4	T5	T6	T7	T8
Ti in T1	−10	1	−1			−21		
Ti in T2	−29	−38		−20	−26			
Ti in T3	−22		−6	−63			−25	
Ti in T4	−48		−43	−9	−46			
Ti in T5		−36		−21		−53		−17
Ti in T6	−25				−16	−13		
Ti in T7 (MFI) ^1^	13	−31	−46	−5				

^1^ Calculated for Ti-MFI-framework with the same methodology as here.

**Table 3:** Calculated electronic and Gibbs free binding energies (BE and ΔG, respectively; in kJ/mol) and Ti-N distances (in pm) in the adsorption complexes of pyridine and acetonitrile at Ti centers incorporated in different crystallographic positions (T1, T2, T3, T4, T5, and T6) of the framework of MSE type zeolite. The calculated IR frequencies (ν_calc_ in cm^−1^) in the range 1400–1600 cm^−1^ for the pyridine complexes and the C-N vibrational frequency for the acetonitrile complexes are also presented, as well as the change of these frequencies with respect to the corresponding values calculated for the gas phase molecules (Δν = ν_calc_(complex) − ν_calc_(gas phase)) in cm^−1^).

	BE	ΔG	Ti-N	ν_calc_	ν_calc_	ν_calc_	ν_calc_	Δν	Δν	Δν	Δν
Pyridine				1567	1565	1454	1420				
T1_pyr	−101	−65	239	1604	1562	1471	1425	37	−3	17	5
T2_pyr	−101	−64	235	1590	1574	1468	1431	23	9	14	11
T3_pyr	−95	−55	239	1607	1568	1471	1424	40	3	17	4
T4_pyr	−95	−61	237	1589	1571	1469	1431	22	6	15	11
T5_pyr	−103	−63	235	1591	1568	1472	1433	24	3	18	13
T6_pyr	−124	−87	232	1611	1561	1466	1427	44	−4	12	7
T6_pyr_vac	−121	−85	234	1606	1564	1464	1426	39	−1	10	6
CH_3_CN				2297							
T1_CH_3_CN	−60	−31	239	2335				38			
T2_CH_3_CN	−52	−24	245	2325				28			
T3_CH_3_CN	−60	−34	239	2327				30			
T4_CH_3_CN	−49	−24	241	2331				34			
T5_CH_3_CN	−67	−33	237	2338				41			
T6_CH_3_CN	−73	−40	231	2341				44			
T6_CH_3_CN_vac	−45	−16	234	2341				44			

## Data Availability

The data presented in this study are available on request from the corresponding author.

## Sample Availability

Additional information related to the calculations is available from the authors.
